# Relationship between Stage of Diabetic Retinopathy and Pulse Wave Velocity in Japanese Patients with Type 2 Diabetes

**DOI:** 10.1155/2013/193514

**Published:** 2013-03-20

**Authors:** Kumiko Tanaka, Toshihide Kawai, Yoshifumi Saisho, Shu Meguro, Kana Harada, Yuka Satoh, Kaori Kobayashi, Kei Mizushima, Takayuki Abe, Hiroshi Itoh

**Affiliations:** ^1^Department of Internal Medicine, School of Medicine, Keio University, 35 Shinanomachi, Shinjuku-ku, Tokyo 160-8582, Japan; ^2^Center for Clinical Research, School of Medicine, Keio University, 35 Shinanomachi, Shinjuku-ku, Tokyo 160-8582, Japan

## Abstract

*Objectives*. We investigated the relationship between the stage of diabetic retinopathy and pulse wave velocity (PWV). *Methods*. This was a cross-sectional study of 689 patients (406 men and 283 women) with type 2 diabetes who were admitted to our hospital from 2004 to 2007. Brachial-ankle pulse wave velocity (baPWV) was measured by an arterial pressure measurement device as PWV/ABI. Diagnosis of diabetic retinopathy was made by ophthalmologists based on the Davis classification: no diabetic retinopathy (NDR), simple retinopathy (SDR), pre-proliferative retinopathy (pre-PDR), and proliferative retinopathy (PDR). *Results*. There was a significant difference in PWV between patients without diabetic retinopathy (1657.0 ± 417.9 m/s (mean ± SD)) and with diabetic retinopathy (1847.1 ± 423.9 m/s) (*P* < 0.001). In addition, the stage of diabetic retinopathy was associated with aortic PWV (1657.0 ± 417.9 m/s in NDR (*n* = 420), 1819.4 ± 430.3 m/s in SDR (*n* = 152), 1862.1 ± 394.0 m/s in pre-PDR (*n* = 54), and 1901.1 ± 433.5 m/s in PDR (*n* = 63) (*P* < 0.001)). *Conclusions*. In patients with diabetic retinopathy, even in those with SDR, PWV was higher than that in patients without diabetic retinopathy. Physicians should therefore pay attention to the value of PWV and macroangiopathy regardless of the stage of diabetic retinopathy.

## 1. Introduction

Pulse wave velocity (PWV) has been used as a noninvasive clinical index of aortic stiffness. It is reported that PWV of patients with diabetes is higher than that of healthy subjects [1]. In a Japanese report of more than 10,000 healthy subjects (age 30 to 74 years), it is reported that the mean ± standard deviation values of PWV are 1331.0 ± 242.0 m/s in male and 1207.0 ± 245.0 m/s in female [2]. It is considered that chronic hyperglycemia causes the progression of arterial stiffness. Chronic hyperglycemia also causes progression of diabetic microangiopathy including diabetic retinopathy. Previous studies have shown that two-hour plasma glucose, glycated hemoglobin, and fasting plasma glucose concentrations are predictors of the development of retinopathy and nephropathy [3, 4]. It was reported that the association of hyperglycemia with retinopathy is stronger than that with nephropathy [3]. In addition, microangiopathy is a strong predictor of the development of the more serious long-term complications of diabetes such as blindness, end-stage renal disease, amputation [5], and cardiovascular disease [6]. Previous studies have shown that PWV, a marker of arterial stiffness, is associated with the presence of diabetic retinopathy [7–11]. 

Retinal capillary microaneurysms are the hallmark of diabetic retinopathy and its earliest reliable sign, and individual acellular capillaries are usually visible histologically in the earliest stages of diabetic retinopathy. As retinopathy becomes more severe, larger patches of acellular capillaries are seen. When lesions like cotton-wool spots, intraretinal microvascular abnormalities, venous beading, and retinal hemorrhages are prominent, diabetic retinopathy is considered pre-proliferative, and new vessels are likely to appear soon on the surface of the retina or optic disc. When new vessels appear on the surface of the retina or optic disc, diabetic retinopathy is said to have entered the proliferative stage [12].

To our knowledge, no study has compared PWV with the stage of diabetic retinopathy. Therefore, we investigated the relationship between increased PWV and the stage of diabetic retinopathy.

## 2. Methods

### 2.1. Subjects

 From January 2004 to December 2007, 732 Japanese patients with type 2 diabetes who were admitted to Keio University Hospital (Tokyo, Japan) were consecutively observed. Among them, 43 patients with acute illness (e.g., cardiovascular event, stroke, infection, etc.) were excluded from the evaluation. Consequently, a total of 689 patients with type 2 diabetes who were admitted due to having poor glycemic control were enrolled in this study. All of their purposes of admission were to control glucose metabolism and education for diabetes. The study protocol was approved by the ethical committee of the hospital. Informed consent was obtained from all patients. 

### 2.2. Measurements

The diagnosis of diabetic retinopathy was made by ophthalmologists based on the Davis classification: no diabetic retinopathy (NDR); simple retinopathy (SDR); pre-proliferative retinopathy (pre-PDR); and proliferative retinopathy (PDR). 

During hospitalization, fasting plasma glucose (FPG), 2-hour plasma glucose (PG), C-peptide (CPR), hemoglobin A1c (HbA1c), glycated albumin (GA), total cholesterol (TC), high-density lipoprotein cholesterol (HDL-C), low-density lipoprotein cholesterol (LDL-C), triglyceride (TG), aspartate aminotransferase (AST), alanine aminotransferase (ALT), urea nitrogen (UN), creatinine (Cr), uric acid (UA) in blood, and 24-hour urine microalbumin were measured. HbA1c was determined by high-performance liquid chromatography (Toso, Tokyo, Japan) and presented as the equivalent National Glycohemoglobin Standardization Program (NGSP) value [13]. Furthermore, we measured systolic/diastolic blood pressure, height, weight, BMI, waist, and hip circumference.

Measurements of brachial-ankle PWV (baPWV) were carried out using an automatic waveform analyzer (Colin Medical Technology Corporation, Japan). Patients lay in the supine position during the test, and occlusion and monitoring cuffs were placed around both the upper and lower extremities. PWV was calculated using the formula: baPWV = (*D*1 − *D*2)/*T*1, where *D*1 is the distance from the heart to the left ankle, and *D*2 is the distance from the heart to the right upper arm. These distances were calculated automatically on the basis of the patient's height. The pressure waveforms obtained at two different sites were simultaneously recorded, and the time interval between the initial rise in the brachial and tibial pressure waveforms was determined as *T*1. ABI was calculated using the formula ABI = ankle systolic BP/brachial systolic BP.

### 2.3. Statistical Analysis

Demographic factors and baseline characteristics were summarized by diabetic retinopathy (DR) and NDR groups. They were compared between the DR and NDR groups using Mann-Whitney *U* test. Next, the patients were divided into four groups according to the stage of diabetic retinopathy (NDR, SDR, pre-PDR, or PDR) to investigate the relationship between each stage and the value of PWV by the Kruskal-Wallis test. The relationship between the PWV and each factor was evaluated with Spearman's correlation coefficient. The selected variables, which were statistically significant and clinically important, were included in nonparametric multiple regression models to evaluate the association between PWV and each stage of diabetic retinopathy adjusted for some covariates. The purpose of these multivariate analyses was to show the robustness of the results from the univariate analysis.

Data are presented as mean ± standard deviation (SD) in the text and tables. The significance level for all tests was two-sided, at 5%. All analyses were performed using SPSS 17.0 (SPSS; Chicago, IL, USA) and SAS 9.2 (SAS; Cary, NC, USA).

## 3. Results

Demographic factors and clinical baseline characteristics of patients are shown in Table 1. Among patients, durations of diabetes, age, SBP, and PWV were significantly higher in patients with retinopathy than in those without.

**Figure 1:**
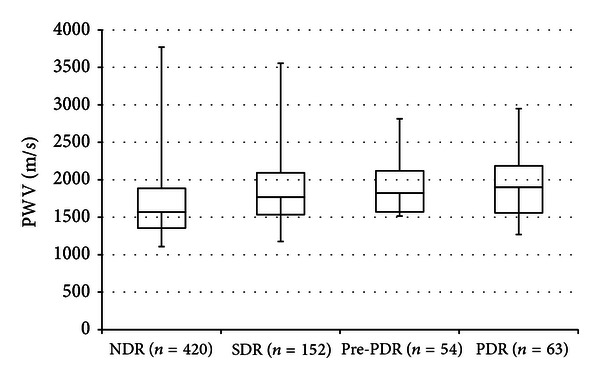
Pulse wave velocity (PWV) in NDR (*n* = 420), SDR (*n* = 152), pre-PDR (*n* = 54), and PDR (*n* = 63). Significant difference among groups (*P* < 0.001) was detected by the Kruskal-Wallis test. NDR: no diabetic retinopathy, SDR: simple diabetic retinopathy, pre-PDR: pre-proliferative diabetic retinopathy, and PDR: proliferative diabetic retinopathy.

PWV in patients with diabetic retinopathy (1847.1 ± 423.9 m/s) was significantly higher than that in patients without diabetic retinopathy (1657.0 ± 417.9 m/s) (*P* < 0.001). Furthermore, there was a significant positive association between the stage of diabetic retinopathy and PWV. PWV was 1657.0 ± 417.9 m/s in NDR (*n* = 420), 1819.4 ± 430.3 m/s in SDR (*n* = 152), 1862.1 ± 394.0 m/s in pre-PDR (*n* = 54), and 1901.1 ± 433.5 m/s in PDR (*n* = 63) (*P* < 0.001) (Figure 1).

Some sensitivity analyses were performed to evaluate robustness of the results from the univariate analysis. Factors significantly correlated with the PWV by means of Spearman's correlation coefficient were BMI, age, SBP, FPG, and HbA1c (Table 2). However, we decided that FPG and HbA1c should not be included in the multivariate analysis because they fluctuate by control of diabetes. We evaluated the association between the stage of diabetic retinopathy and PWV adjusted for the above-mentioned covariates by using nonparametric multiple regression analyses. As a result, when taking account of the covariates that have an effect on PWV, PWV tended to increase as the stage of diabetic retinopathy progression (*P* < 0.001).

## 4. Discussion

Measurement of aortic PWV is considered the gold-standard evaluation of arterial stiffness [14]. Values of PWV in patients with diabetes are higher than those in healthy people in the same generation [15]. The prevalence of arterial stiffness is increased in patients with type 2 diabetes, and these patients are at particularly higher risk for cardiovascular morbidity and mortality. Several studies have shown that diabetic retinopathy is associated with cardiovascular complications [16–18].

In the present study, PWV was significantly higher in patients with diabetic retinopathy than in those without. This finding supports the report that diabetic retinopathy is the microvascular complication with the strongest association with increased aortic stiffness [7]. In addition, there was a relationship between PWV and stage of diabetic retinopathy in Japanese patients with type 2 diabetes, in our study. We showed that the values of PWV in patients with diabetic retinopathy, even in those with SDR, were higher than those in patients without diabetic retinopathy. Henricsson et al. reported that the severity of diabetic retinopathy might be associated with survival, primarily owing to cardiovascular death in patients with diabetes [19]. While the severity of diabetic retinopathy might be important for prediction of macroangiopathy, physicians should pay more attention to macroangiopathy in patients with diabetic retinopathy, regardless of the stage. 

Increased arterial stiffness is thought to be related to not only hyperglycemia but also to carbonyl and oxidative stress, chronic inflammation, endothelial dysfunction, and formation of advanced glycation end products (AGEs) [7]. It is reported that PWV is associated with the duration of diabetes and with the accumulation of fluorescent AGEs [20]. Besides, several reports indicate that the stage of diabetic retinopathy correlates with the accumulation of AGEs [21, 22]. Therefore, there is a possibility that the stage of diabetic retinopathy is associated with PWV through the accumulation of AGEs.

Several limitations should be taken into account when considering the results of this study. First, the cross-sectional study design and small sample size for each stage of diabetic retinopathy in our study make it difficult to infer the association between PWV and retinopathy. Second, we could not consider the effects of prescribed medication, for instance, anti-platelet agents, which could influence the state of both retinopathy and arterial stiffness. Third, the raw data might have deviated slightly because there was more than one PWV technician and ophthalmologist. However, it was thought that the influence of bias was small because the technicians and ophthalmologists were experts and were not aware of this study when they carried out the examinations. Lastly, the patients with type 1 diabetes were not included in this study. In a recent meta-analysis of observational studies, diabetic retinopathy predicted all-cause mortality and cardiovascular events in patients with type 2 diabetes and also type 1 diabetes [18]. Based on these findings, physicians should pay attention to latent macroangiopathy in patients with not only type 2 diabetes but also type 1 diabetes who have diabetic retinopathy, even SDR.

In conclusion, this study suggested that PWV is significantly higher in patients with diabetic retinopathy than in those without, and that there is a relationship between the stage of diabetic retinopathy and PWV in Japanese patients with type 2 diabetes. Physicians should pay attention to latent macroangiopathy in patients with type 2 diabetes who have diabetic retinopathy, even SDR.

## Figures and Tables

**Table 1:** Clinical characteristics of patients with type 2 diabetes mellitus.

	Total	NDR	DR	*P* value
*N* (male/female)	689 (406/283)	420	269	
Duration (years)	12.0 ± 10.0	10.0 ± 9.5	16.0 ± 10.0	<0.001
Age (years)	62.2 ± 13.4	61.0 ± 14.3	65.0 ± 11.6	0.002
BMI (kg/m^2^)	25.0 ± 5.5	25.6 ± 5.8	24.3 ± 4.7	0.001
SBP (mmHg)	132.0 ± 20.9	130.0 ± 20.0	135.0 ± 22.0	0.009
DBP (mmHg)	76.9 ± 14.0	77.0 ± 13.6	76.0 ± 14.5	0.907
HbA1c (%)	9.6 ± 2.0	9.7 ± 2.2	9.5 ± 1.7	0.233
LDL-C (mg/dL)	127.2 ± 37.1	128.0 ± 37.2	125.9 ± 37.2	0.295
PWV (m/s)	1731.2 ± 430.0	1657.0 ± 417.9	1847.1 ± 423.9	<0.001

Data are shown as mean ± SD. Comparison between patients without diabetic retinopathy (NDR) and with DR by Mann-Whitney's *U* test.

BMI: body mass index, SBP: systolic blood pressure, DBP: diastolic blood pressure, LDL-C: low-density lipoprotein cholesterol, and PWV: pulse wave velocity.

HbA1c is presented as the National Glycohemoglobin Standardization Program (NGSP) value.

**Table 2:** Relationship between PWV and clinical factors by Spearman's correlation.

	Correlation coefficient	*P* value
BMI (kg/m^2^)	−0.19	<0.001
Age (years)	0.61	<0.001
SBP (mmHg)	0.35	<0.001
DBP (mmHg)	−0.01	0.73
FPG (mg/dL)	−0.14	<0.001
HbA1c (%)	−0.20	<0.001
LDL-C (mg/dL)	−0.05	0.20
HDL-C (mg/dL)	0.00	0.98
TG (mg/dL)	−0.01	0.73
TC (mg/dL)	−0.04	0.33
ABI	0.07	0.06

BMI: body mass index, SBP: systolic blood pressure, DBP: diastolic blood pressure, FPG: fasting plasma glucose, LDL-C: low-density lipoprotein cholesterol, HDL-C: high-density lipoprotein cholesterol, TG: triglyceride, TC: total cholesterol, and ABI: ankle brachial index.
